# Teamwork: relevance and interdependence of interprofessional education

**DOI:** 10.1590/S1518-8787.2017051006816

**Published:** 2017-04-18

**Authors:** M Tamayo, A Besoaín-Saldaña, M Aguirre, J Leiva

**Affiliations:** I Departamento de Kinesiología. Facultad de Medicina. Universidad de Chile. Santiago, Chile

**Keywords:** Students, Health Occupations, Health Knowledge, Attitudes, Practice, Social Perception, Patient Care Team, Interdisciplinary Communication, Interprofessional Relations

## Abstract

**OBJECTIVE:**

Determine the perception of university students regarding interprofessional and interdependent work between team members in their inclusion in primary care.

**METHODS:**

Analytical cross-sectional study. The sampling had a probabilistic, stratified random type with 95% confidence and 5% margin of error. Seven-hundred and four students of Public Universities in Santiago (Chile) answered self-administered questionnaire.

**RESULTS:**

Ninety-seven point eight of students say that interprofessional work is important; 27.1% of them declare that their university did not seem to show that their study plans were important. The professionals listed as most important in teams are physicians and nurses.

**CONCLUSIONS:**

Spaces for development and institutional support are key elements to promote interprofessional work. If this competence can involve each academic unit in their different formative spaces there will be a significant contribution to said promotion. Teamwork is a pending task.

## RESUMEN

**OBJETIVO:**

Determinar la percepción de estudiantes universitarios respecto al trabajo interprofesional e interdependencia entre los miembros del equipo en su inserción en la atención primaria.

**MÉTODOS:**

Estudio de tipo analítico y transversal. El muestreo fue de tipo aleatorio, probabilístico estratificado con un 95% de confianza y un 5% de margen de error. Se utilizó un cuestionario auto-administrado en 704 estudiantes de Universidades del Estado en Santiago de Chile.

**RESULTADOS:**

Un 97,8% de los estudiantes opinan que el trabajo interprofesional es importante; un 27,1% de ellos declara que su universidad no le ha entregado importancia en sus planes de estudios. Los profesionales mencionados como más importantes en el equipo son los médicos y enfermeras.

**CONCLUSIONES:**

Espacios de desarrollo y respaldo institucional son elementos claves para promover el trabajo interprofesional. Que esta competencia logre involucrar a cada unidad académica en sus diferentes espacios formativos será un aporte significativo en aquello. Trabajo en equipo es una tarea pendiente.

## INTRODUCTION

Historically, the focus of medical education has been the individual performance of health professionals. However, given the changes in focus, structure, and needs of healthcare systems, the focus has changed to teamwork^1^. This concept implies a coordinated action, led by two or more individuals, which implies mutually agreed goals, and requires clear understanding and respect of the roles and functions of each member. Teamwork, rather than an end, is a process and it requires the skill to work as colleagues instead of as superior-subordinate^2^.

In this sense, the common work performed by professionals of different categories is multi-professionalism, in which their individual contribution results in a final product that meets the unique needs of each party in the solution of the problems identified^3^. Both the collective construction of the work that will be carried out by a multidisciplinary team and the possibilities of incorporating attention to professional practices, indicate the need to identify and develop communicative dimensions in the subjects involved in the care (workers and users). The multidisciplinary teams must overcome the agreements and articulations centered only in the relationships. The World Health Organization^5^ notes that collaborative practice optimizes the results of health care by delivering a comprehensive care to patients, their families, caregivers, and communities. Professionals prepared for collaborative practice learned to work in an interprofessional team through effective training in interprofessional education. This education occurs when students from two or more professions learn about, of and between themselves to allow an effective collaboration^5^.

In the world and in Chile, the basis of the health care model is strengthening the primary health care (PHC)^6^, in which the teams are an essential part. On the other hand, the Pan American Health Organization (PAHO) stipulates that the belonging States must advance when it comes to “ensuring the development of the human talent required to put PHC into practice successfully with the incorporation of multidisciplinary teams” (p.82)^7^.

The human resources development implies a technical and professional training, and it must include the team approach. It is not possible to ensure the effectiveness of the team only by training its members individually in health care, but one must also consider the process of direction and monitoring of the team as a whole, and how the team faces and solves problems^2^.

The evidence shows that the skills to work in teams and collaboratively are not intuitive and are not learned by doing the job itself^8^ ^,^ ^9^. This could justify that the acquisition of such skills must occur during academic training. On the other hand, although the curricular activities of the various academic programs include common courses, generally, there are no interaction points between them, as there is a lack of “interprofessional education”^10^. In addition, one of the main problems is the lack of knowledge of each team member’s roles, powered by prejudices or negative stereotypes that one has of the professions at the time of admission. For this reason, we propose that interprofessional education “be incorporated among the specific activities of each discipline”(p.265)^3^ and the curriculum^11^.

Currently, the WHO says that the education and health systems must work together to coordinate strategies for human resources in health. If the human resources plan in health and the policies they formulate are integrated, interprofessional education and collaborative practices can be sustained^5^.

On the other hand, the problem derived from the weakness in the employment relationships of the teams has a direct influence; therefore, to give more stability to the professional can mean an increased possibility of accumulation of knowledge and skills, resulting in greater consistency and durability in the training of future professionals and in family health^12^.

For these reasons, in Chile, different educational institutions include in their training profiles the ability to work in health teams in health careers^13^ as a gesture of consistency with the current milestones that define the training of health professionals. On the other hand, this mainstreaming of teamwork in health career training is responsible for the homogenization in the curricula, after implementation of the accreditation process in different careers. It is “the process of analysis of existing mechanisms within the autonomous institutions of higher education to ensure their quality, taking into consideration both the existence of these mechanisms and their application and results (art. 1)”^a^. This process is part of the mechanisms to regulate the supply of training centers that offer careers in health and most of them are private, not dependent on the Council of Deans, and not accredited, which is a clear deregulation of production quality in health professionals^14^.

The objective of this research was to determine the perception of university students regarding interprofessional and interdependent work between team members in their inclusion in primary care.

## METHODS

**Figure 1:**
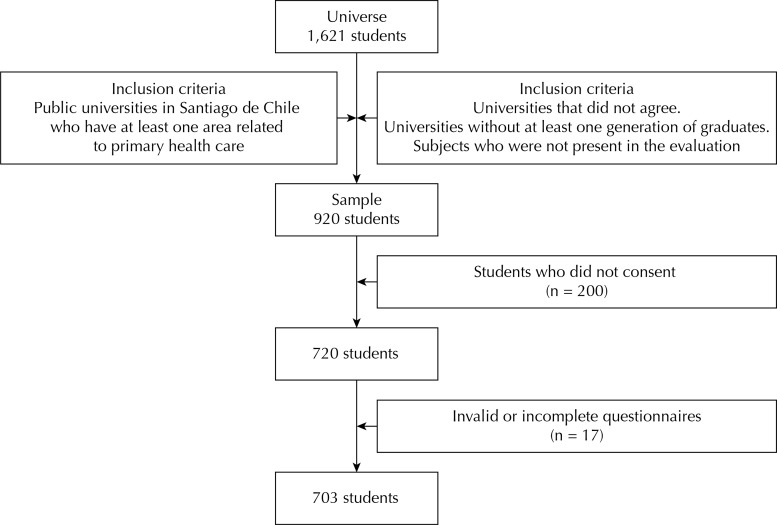
The sample selection process, with the criteria of inclusion, exclusion, and steps of sampling.

Analytical and cross-sectional study. The sampling had a probabilistic, stratified random type with 95% confidence and 5% margin of error. The universe consists of all students of the level before the residency or clinical practice during the year of 2012 in professional careers developed in primary care belonging to public universities in the metropolitan region of Chile. The universe consisted of 1,621 students. The occupations selected were: Medicine, Chemistry and Pharmacy, Dentistry, Midwifery, Nursing, Nutrition and Dietetics, Physical Therapy, Medical Technology, Social Work, Psychology, Occupational Therapy and Speech Therapy. When applying the exclusion criteria, the sample diminished to 704 students (Figure 1), complying with the lower limit to the level of representation attributed.

The basis to the questionnaire was specific literature about collaborative and interprofessional work^8^ ^,^ ^10^ ^,^ ^12^ ^,^ ^15^.

Subsequently, the health education experts or members of the Department of Education in Health Sciences of the Medical School of Chile University performed a content and concept validation. This part was finished with a test, in which the students of a course with similar characteristics to the sample, but who are not included in this study, took a questionnaire correcting clarity, accuracy, and validity of the instrument.

Each student received a self-administered questionnaire to establish the importance given by the universities to interprofessional work, the need for interdisciplinary work and the importance given to teamwork in the areas of primary, secondary and tertiary health care. For those questions, we used the Likert scale of five alternatives. On the other hand, information on the knowledge, indispensability, and interdependence with the different health careers was necessary. Of the total valid research (n = 703), in the questions about the value, importance, and knowledge of students about careers, the student’s answers about the careers that they were studying were excluded, with talus goal to reduce the over-representation bias of their opinions.

The program SPSS 19.0 for Windows analyzed the data by frequency analysis and measures of central tendency. To determine differences, we applied the Chi-square test with a significance level lower than 0.05.

This study had the approval of the Committee on Ethics on Human Research of the University of Chile’s School of Medicine, which considered for approval the principles of the Declaration of Helsinki, the International Guide to Ethics for Biomedical Research involving human subjects, the 1992 CIOMS, and the 1996 ICH Guidelines Clinical Practice. All participants who completed the questionnaire had previously signed an Informed Consent Form (ICF).

## RESULTS

The sample of 704 health careers students (63.8% women) had a median age of 22 years and an interquartile range of one.

Table 1 describes the sample, differentiated by gender, regarding the previous experience of students in the PHC and the academic program to which the students surveyed belonged.

**Table 1:** Sample description, differentiated by gender.

Variable		Male		Female		Total	
		
		n	%	n	%	n	%
Attendance in primary health care	With no experience in primary health care	135	32.8	276	67.2	411	58.5
	With experience in primary health care	120	41.1	172	58.9	292	41.5
Profession	Midwifery	4	14.8	23	85.2	27	3.8
	Occupational therapy	4	17.4	19	82.6	23	3.3
	Nursing	26	17.9	119	82.1	145	20.6
	Nutrition and dietetics	8	20	32	80.0	40	5.7
	Speech therapy	8	25	24	75.0	32	4.6
	Psychology	15	25.9	43	74.1	58	8.3
	Social Work	5	27.8	13	72.2	18	2.6
	Chemistry and pharmacy	12	34.3	23	65.7	35	5.0
	Dentistry	28	35.4	51	64.6	79	11.2
	Physical Therapy	22	51.2	20	47.6	42	6.0
	Medicine	108	59.3	74	40.7	182	25.9
	Medical technology	15	68.2	7	31.8	22	3.1

**Figure 2:**
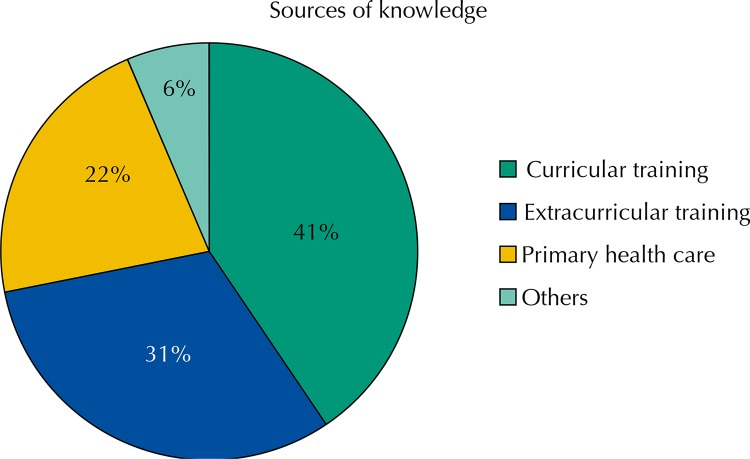
University students’ source of knowledge on health team members in primary care, 2013. (N = 679)

Taking into account that the knowledge acquired in other professions can have different origins, students were required to distribute this source in different categories. Figure 2 shows a predominance of curricular training in obtaining this knowledge in 40.5% of the weight of training in this area.

Most students claimed to agree that their educational institutions give great importance to interprofessional work; however, 27.1% of respondents considered the opposite.

Table 2 expresses the differences by gender in appreciation of the importance, necessity, and indispensability of training for interprofessional work. There is an agreement to recognize that the University has devoted much importance within the curriculum the interprofessional work on HCP and that to dedicate a good primary-level health care, interprofessional work is desirable, but not essential, which presents a significant difference between men and women. It is possible to observe the previous information given off by professional career in Table 3.

**Table 2:** The difference by gender in appreciation of the importance, necessity, and indispensability of training in interprofessional work.

Question	Answer	Male		Female		Total		p
		
		Recount	%	Recount	%	Recount	%	
Question 1. The university dedicated, within the curriculum, great importance to interprofessional work in primary health care.	Disagree	76	36.0	87	22.3	163	27.1	0.001*
	Agree	135	64.0	303	77.7	438	72.9	
	Total	211	100	390	100	601	100	
Question 2. For a good care in the integral health care model, interprofessional work is necessary.	Disagree	9	3.6	6	1.3	15	2.2	0.059
	Agree	241	96.4	439	98.7	680	97.8	
	Total	250	100	445	100	695	100	
Question 3. In order to dedicate a good primary-level health care, interprofessional work is desirable, but not essential.	Disagree	168	77.8	346	84.8	514	82.4	0.035*
	Agree	48	22.2	62	15.2	110	17.6	
	Total	216	100	408	100	624	100	
Question 4. In order to dedicate a good secondary and tertiary-level health care, interprofessional work is desirable, but not essential.	Disagree	190	86.0	366	90.2	556	88.7	0.115
	Agree	31	14.0	40	9.8	71	11.3	
	Total	221	100	406	100	627	100	

* Significant difference (p < 0.05).

**Table 3:** The difference by area in appreciation of the importance, necessity, and indispensability of training in interprofessional work.

Variable	The university dedicated, within the curriculum, great importance to interprofessional work in HCP		For a good care in the integral health care model, interprofessional work is necessary		In order to dedicate a good primary-level health care, interprofessional work is desirable, but not essential		In order to dedicate a good secondary and tertiary level health care, interprofessional work is desirable, but not essential	
			
	Disagree	Agree	Disagree	Agree	Disagree	Agree	Disagree	Agree
							
	n (%)	n (%)	n (%)	n (%)	n (%)	n (%)	n (%)	n (%)
Social work	2 (12.5)	14 (87.5)	0 (0)	18 (100)	14 (87.5)	2 (12.5)	14 (93.3)	1 (6.7)
Nursing	13 (9.7)	121 (90,3)	6 (4.1)	139 (95.9)	120 (90.2)	13 (9.8)	127 (92.7)	10 (7.3)
Speech therapy	8 (33.3)	16 (66.7)	0 (0)	32 (100)	21 (80.8)	5 (19.2)	27 (90.0)	3 (10.0)
Physical therapy	2 (5.1)	37 (94.9)	0 (0)	42 (100)	31 (83.8)	6 (16.2)	34 (89.5)	4 (10.5)
Midwifery	1 (3.7)	26 (96.3)	1 (3.7)	26 (96.3)	22 (88.0)	3 (12.0)	20 (80.0)	5 (20.0)
Medicine	53 (35.1)	98 (64.9)	4 (2.2)	175 (97.8)	128 (80.0)	32 (20.0)	135 (83.9)	26 (16.1)
Nutrition	4 (11.4)	31 (88.6)	2 (5.0)	38 (95.0)	33 (86.8)	5 (13.2)	32 (86.5)	5 (13.5)
Dentistry	18 (28.1)	46 (71.9)	1 (1.3)	77 (98.7)	44 (69.8)	19 (30.2)	63 (91.3)	6 (8.7)
Psychology	22 (45.8)	26 (54.2)	0 (0)	56 (100)	43 (78.2)	12 (21.8)	37 (86.0)	6 (14.0)
Chemistry and pharmacy	18 (72.0)	7 (28.0)	1 (2.9)	34 (97.1)	26 (81.3)	6 (18.8)	28 (87.5)	4 (12.5)
Medical technology	15 (78.9)	4 (21.1)	0 (0)	20 (100)	13 (72.2)	5 (27.8)	18 (94.7)	1 (5.3)
Occupational therapy	7 (36.8)	12 (63.2)	0 (0)	23 (100)	19 (90.5)	2 (9.5)	21 (100)	0 (0)

Total	163 (27.1)	438 (72.9)	15 (2.2)	680 (97.8)	514 (82.4)	110 (17.6)	556 (88.7)	71 (11.3)

**Figure 3:**
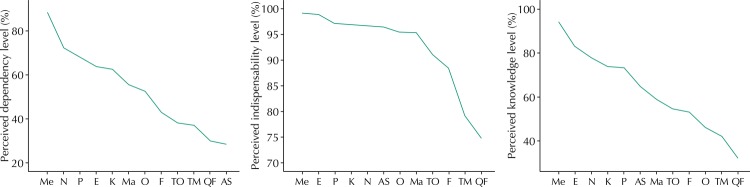
Perception of the level of knowledge, indispensability, and dependency for different health professions.

Figure 3 represents the perception of the level of knowledge, indispensability, and dependency for different health professions. On the other hand, each part is divided by the average; thus, the occupations that are on the line have better evaluation than those that fall under it. This question had a disclaimer asking the respondent to consider the context of primary care.

Ma: Midwifery; Me: Medicine; N: Nutrition; P: Psychology; E: Nursing; F: Speech Therapy; K: Physical Therapy; O: Dentistry; TO: Occupational Therapy; TM: Medical Technology; QF: Chemistry and Pharmacy; AS: Social Work

## DISCUSSION

This study describes the perceptions of students in professional careers present in Primary Health Care in Chile, associated to interdependence, indispensability, and level of knowledge they had about the other professionals, and how those experiences affect variable experiences like gender or previous experience with this sector. The main findings identified were the high level of knowledge, indispensability, and interdependence that the classical structure of medical staff (doctors-nurses) has in regards to the HCP healthcare team. Students exposed to this knowledge and perceptions based it primarily on their experience within the curriculum of their careers. In addition, women give more relevance to the non-renunciation and to knowledge about interprofessional work in their education before graduation.

In general, the basis for the study of interprofessional work in the past five years were specific areas, such as dentistry^15^, diabetes^16^, traumatology^17^ and cancer^18^. However, studies aimed at analyzing aspects linked to education for health students, i.e., of skills needed for performance in interprofessional teams, are scarce and do not show evidence of their positive results^19^ ^,^ ^20^. One of the explanatory aspects of this situation possibly relates to the high heterogeneity of expressions that supports interprofessional work. Although institutional efforts for contribution to the development of multi-professional teams are desirable and necessary, because they are an ideological posture for the development of their future graduates, the health center’s efforts should encourage and give new meanings to these practices so that they feedback and foster integral care that promotes people’s autonomy and equity in health.

These results are responsible for the challenges in the development of teamwork skills in students before graduation, a key element for the functioning of a health system. These skills are more critical on HCP, where the education of human resources remains an incomplete challenge in their entirety^7^. Teamwork is very important to provide full assistance to the patient and their family. Therefore, when all members are aware of the families’ necessities, the focus is total and more efficient, since the whole team participates in the solution of the problem^21^.

Teamwork occurs in the context of concrete practice, where hierarchical relationships between doctors, not doctors and different levels of subordination happen along with a flexible division of labor and technical autonomy with interdependence. Therefore, it is possible to build an integrated team, even in situations where asymmetric relations occur between the different professionals. Through communicative action, the debate on technical assistance and the uneven social value of work areas lead to different levels of integration. This presupposes not only to share technical premises but also, and especially, an ethical horizon^22^.

Within this context, deepening the knowledge of the education of future professionals who will dedicate services to people is of vital importance to understand the current state of this commitment and its projection in the community. We believe that a thorough review of the formative processes, as well as the importance attached, is of the utmost importance to change the conducts and potentiation of interprofessional work in health.

On the other hand, if we separate our results according to the program of study to which they belong, a greater concentration of students who say they do not realize the importance of interprofessional work in their study plan o in the areas of Chemistry and Pharmacy and Medical Technology. On the other hand, in areas like Psychology, Occupational Therapy, Medicine, Speech Therapy and Dentistry, only 20% of students believe the same. This pattern is key, given the importance of this skill in health human resource. We can conclude that the development of this skill is unequal in different study programs, which implies the existence of professionals with insufficient capacities to enter optimally in health teams. It is worth mentioning that an association has been identified between the answers to question one and question three between men and women, with women being more critical of the level of importance of teamwork training in the curriculum (p < 0.01), and also more discerning when considering teamwork to be essential in the APS (p = 0.035). This is consistent with the findings of studies that determined greater preparation and predisposition of female health student in the development of teamwork^23^ ^,^ ^24^.

The ability to work in interprofessional teams at APS was considered to be expendable by 15.6% of the students. This goes against the claims by international bodies regarding this^2^ ^,^ ^5^ ^,^ ^7^. In addition, the profiles of graduation of the areas analyzed declared this as a feature of their graduates; therefore, this commitment is unfulfilled for the society around it.

Medicine and Nursing were the occupations recognized as the most indispensable when it came to HCP health teams. This happens because these two occupations are a social icon of health care. However, to find out the reason for this greater recognition we must address the topic with another methodology. In this way, strengthening teamwork education must involve acknowledging its component parts, including both cross elements and disciplinary ones, those that allow an integral approach of our populations with the respective curricular expression, element shared by Bicudo et al^3^.

In general, the student’s perception is positive for interprofessional work, showing a predisposition to develop these skills. However, if educational institutions are responsible for ensuring the development of human resources capable of correctly interacting with health team members, dedicating an integral and multidisciplinary health, the absence of actions in this sense puts the fulfillment of this commitment in serious jeopardy. Even more when the work of the multidisciplinary team, the relationship between its members, especially in outpatient treatments day inpatient treatments, denotes that the derivation between professionals was the most common strategy to respond to the various needs of the users^4^. The intersectional work is critical.

Based on the results obtained, it is necessary to deepen the current state of interprofessional education in people who currently work in HCP, and thus analyze the phenomenon of a broader view as long as it is possible to guide the interventions necessary to get improvements in the current system and its results. Following this subject, it is possible to carry out a comparative analysis of what is the real knowledge and skills that future professionals must master to develop in an interprofessional team at any level of care.

Taking into consideration a complex, ecological structure, with multiple interests and positions within the power relations, strengthening education in interprofessional work is useful to adopt more realistic and relevant positions on health education.

One of these positions is to understand that a concept of health education focused on the development of human beings is a theoretical and epistemological option when compared with other options^25^. Despite internal and external constraints on labor performance, it is the worker who develops and sustains the action project in health institutions, groups and daily practice. In addition, in health, the job object is another concrete individual who influences, in a dialectical relationship, the work process of professionals^26^.

Here we must cite Luís Otávio Farías: “the relationships and interactions empirically perceived go further, to the extent that they are also an expression of the broader socio-historical processes” (p.1241)^27^. The academic programs consistent with the training of workers in health must redefine the professional skills, crosscutting and emerging, in order to comply properly with the current social needs. For example, few programs in the region insisting on prevention, health promotion, and primary care^28^.

Multi-professional work offers an interaction between specific and technical knowledge; through this interaction arise new proposals for intervention that only one professional could not make. This is the result of the union of different types of knowledge. The former must be associated with a broad vision of health that incorporates professional skills with a humanistic and integral understanding of the health-disease process taking into consideration the political, economic, educational and familiar aspects, among others^29^. In other words, a multi-professional work with a social determinants perspective.

Teamwork is something desirable to the extent that our efforts focus only on expressing it formally. When having institutional support and development spaces is transcendental. If this competence can involve each academic unit in their different formative spaces, there will be a significant contribution. Teamwork is a pending task.

## Footnotes

a Ministerio de Educación de Chile. Ley nº 20.129, de 23 de octubre de 2006. Establece un sistema nacional de aseguramiento de la calidad de la educación superior. Santiago de Chile; 2006 [cited 2016 Dec 11]. Available from: http://www.supersalud.gob.cl/normativa/668/w3-article-14436.html
